# POLICY SERIES: ADDRESSING LONELINESS AMONG OLDER ADULTS THROUGH RESEARCH, COMMUNITY PROGRAMS, AND A NEW FEDERAL POLICY AGENDA

**DOI:** 10.1093/geroni/igz038.2232

**Published:** 2019-11-08

**Authors:** Erica Solway, Brian W Lindberg

**Affiliations:** 1 Institute for Healthcare Policy and Innovation, University of Michigan, Ann Arbor, Michigan, United States; 2 The Gerontological Society of America, Washington, District of Columbia, United States

## Abstract

Millions of older adults experience feelings of loneliness. A growing body of research has found that chronic loneliness can impact memory, physical well-being, mental health, and life expectancy rivaling the impact on health outcomes of obesity and smoking. Loneliness has been found to impact memory, physical well-being, mental health, and life expectancy. In this session, GSA policy advisor Brian Lindberg will lead a data-driven discussion about who experiences loneliness and isolation and how we might create opportunities for connectedness through new areas of research, forward-thinking policies, and innovative community programs. Presenters include Erica Solway, associate director for the National Poll on Healthy Aging, who will highlight results from a poll conducted in October 2018 among a nationally representative sample of adults age 50 to 80 which found that more than one in three respondents felt a lack of companionships and more than one in four felt socially isolated. Then Catherine Spensley, Director of the Senior Division at Felton Institute, will describe lessons learned in developing and delivering culturally and linguistically appropriate programs and services that foster community and social connections among socially isolated, low income older adults in San Francisco. Finally, Andrew MacPherson, Principal at Healthsperien, LLC, and Director of the Coalition to End Social Isolation & Loneliness will describe stakeholder efforts to advocate for federal legislative and regulatory policy options to address the epidemic including increased funding for and access to supportive services, health care, technology, and public and private research initiatives.

